# Case report: Treatment of anterior cerebral artery aneurysms with combined remodeling technique and flow diverter deployment through a dual lumen balloon catheter

**DOI:** 10.3389/fneur.2022.1058328

**Published:** 2022-12-15

**Authors:** Leonardo Renieri, Francesco Capasso, Sergio Nappini, Antonio Laiso, Carolina Capirossi, Nicola Limbucci

**Affiliations:** Interventional Neurovascular Unit, Careggi University Hospital, Florence, Italy

**Keywords:** distal aneurysms, flow diverter (FD), remodeling technique, endovascular treatment (EVT), difficult aneurysms

## Abstract

We present the technical aspects of embolization for two unruptured medium-sized aneurysms of the anterior cerebral artery treated with balloon-remodeling technique and loose coiling of the sac with the final deployment of a 0. 017-compatible flow diverter. Both procedures were performed with dual antiplatelet therapy premedication and under general anesthesia. The anatomy of the two aneurysms was similar with a wide neck and the presence of a collateral artery branching off it, which required the additional use of a compliant balloon in order to retain patency and avoid coil protrusion. After initial coiling, a nitinol flow-diverter was deployed through a coaxial dual lumen balloon microcatheter. Both these interventions encountered no complications, and the patient was discharged on day 2. At 6-month clinical and radiological follow-up, neither patient had neurological deficits, the aneurysms were both completely occluded, nor the stented arteries were patent along with their collateral branches.

## Background

Flow diverters (FDs) have gained wide acceptance for the treatment of unruptured intracranial aneurysms due to their proven efficacy and safety with high occlusion rates on long-term follow-up. Nevertheless, their profile and stiffness originally limited their use for distal locations.

The Derivo 2 Embolization Device (Acandis, Germany) and Silk Vista Baby (Balt, France) are two of the newest FDs and represent a dramatic improvement in ease of use distally as they accommodate a 0.017 microcatheter ensuring a safe option for aneurysms of small vessels. Their low profile also makes them compatible with dual lumen balloon catheters, representing a safe alternative to braided stents after balloon-assisted coiling.

## Case presentation

Written informed consent was obtained from the individuals for the publication of any potentially identifiable images or data included in this article.

### Case 1

An adult patient with subarachnoid hemorrhage and multiple aneurysms was admitted to our institution and treated endovascularly for the right middle cerebral artery and posterior communicating artery aneurysms.

After 6-months and a full recovery, the patient was initiated on dual antiplatelet therapy and called back for treatment of a 6 × 5 mm aneurysm of the A3 segment of the right anterior cerebral artery. The aneurysm had an irregular shape, a wide neck, and the internal middle frontal cerebral artery arose directly from the sac.

Under general anesthesia, *via* right femoral access, a Neuron Max (Penumbra, USA) and a Navien 0.072 (Medtronic, Ireland) were parked in the right internal carotid artery (ICA). A Scepter C 4 × 10 (MicroVention, USA) balloon was then positioned at the neck of the aneurysm over a Synchro 0.014 guidewire (Stryker, USA). An Echelon 10 (Medtronic, Ireland) microcatheter was then navigated into the aneurysm and a single coil (Stryker 360 Ultra 5 × 10) was deployed while the balloon was inflated. At the end of coiling, a Derivo 2 2.5 × 10 was deployed covering the neck of the aneurysm through the Scepter. No complications occurred and the patient was discharged on post-procedure day 2.

**Figure 1:**
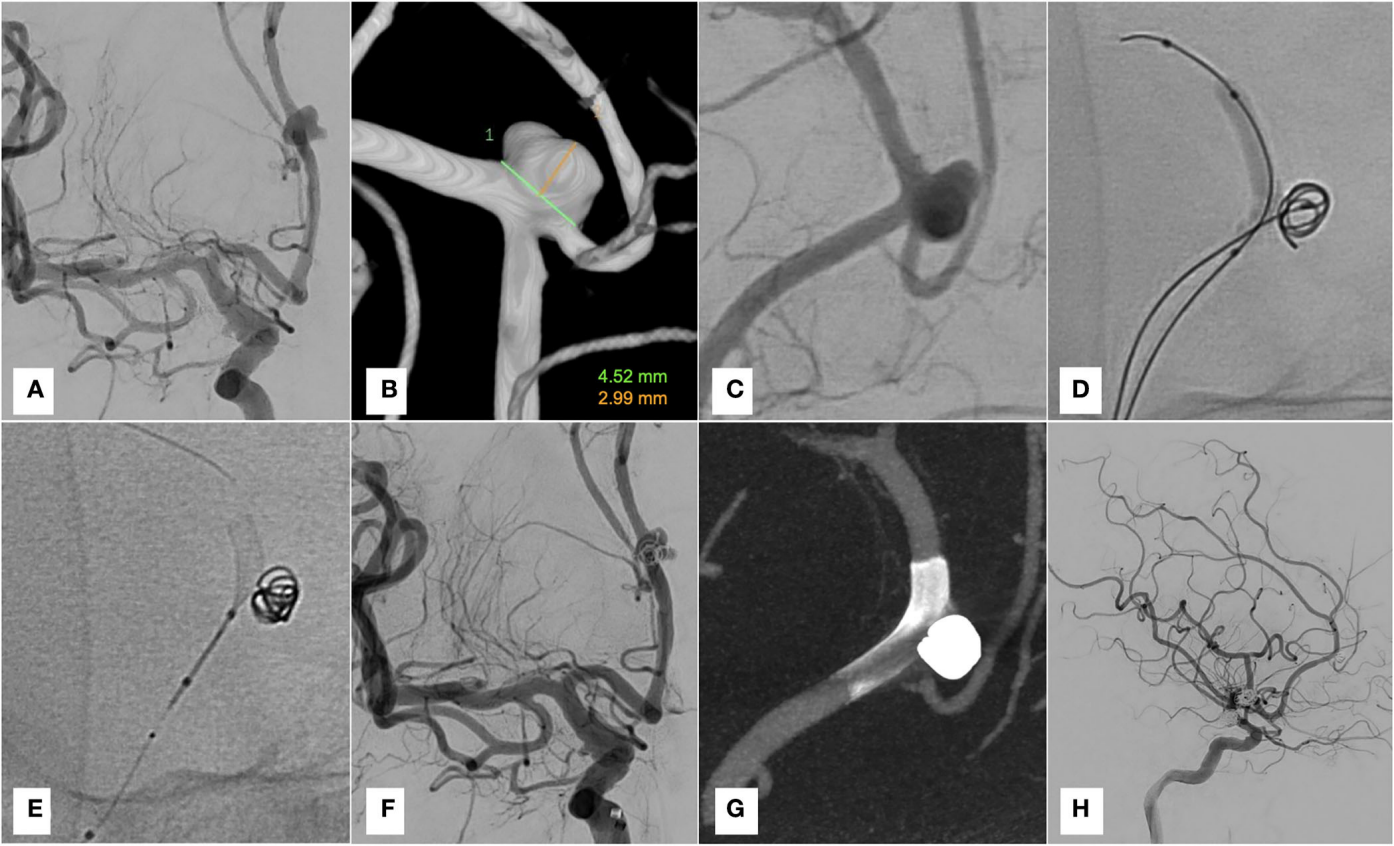
Case 1. **(A–C)** Pre-treatment DSA and 3D reconstruction. **(D)** Coiling with Scepter C inflated. **(E)** Deployment of Derivo 2 through the dual lumen balloon. **(F,G)** Final run and Vaso-CT. **(H)** Six-month-DSA follow-up.

Six-month DSA follow-up demonstrated complete occlusion of the aneurysm with patency of the middle internal frontal artery (Figure 1).

### Case 2

A patient in their 60s' underwent magnetic resonance angiography (MRA) as part of a vertigo workup and an aneurysm was detected on the A3 segment of the left anterior cerebral artery. Pre-treatment digital subtraction angiography (DSA) showed a 7.5 × 4 mm aneurysm at the pericallosal-callosomarginal bifurcation. The neck of the aneurysm was wide and a tiny pericallosal branch arose directly from the lesion.

**Figure 2:**
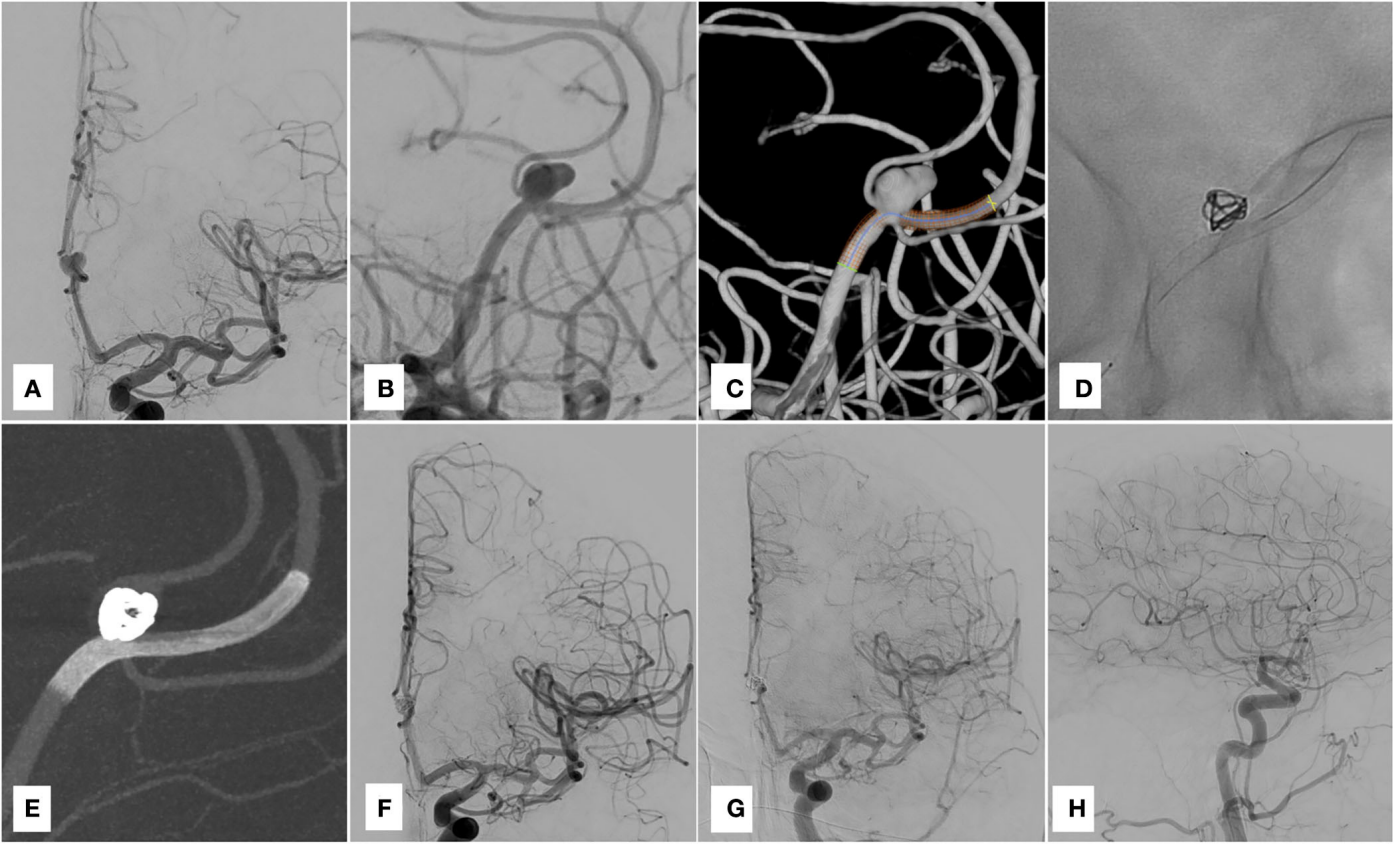
Case 2. **(A–C)** Pre-treatment DSA and 3D reconstruction. **(D)** Derivo 2 deployed. **(E,F)** Post-treatment run and Vaso-CT. **(G,H)** Six-month-DSA follow-up.

Under general anesthesia and with dual antiplatelet premedication, the same materials were employed as in the previous case and a single coil was detached (Stryker 360 Soft 5 × 10) while inflating the balloon. A Derivo 2 2.5 × 15 was then deployed into the calloso-marginal artery with some difficulties related to the trackability of the stent. On the final angiogram, contrast stagnation within the aneurysm was noted. The patient was discharged home on day 2 with no deficits. The 6-month DSA follow-up demonstrated complete occlusion of the aneurysm and patency of the pericallosal artery (Figure 2).

## Discussion

Elective treatment of distal aneurysms with FDs has become feasible due to new generation low-profile stents (1, 2). In most cases, the stent itself is enough to guarantee occlusion of the sac and any conventional microcatheter may be used to deploy it. In bifurcation aneurysms in which the shunted branch has no direct collateral supply, coiling of the sac should be considered and sometimes a compliant balloon might be useful to achieve a good cast of the coils preserving the branching artery (3). Until now, if balloon-assisted coiling had to be performed, either an exchange maneuver or a second catheterization had to be carried out as FDs were not compatible with dual lumen balloon catheters.

However, Silk Vista Baby and Derivo 2 are now available and accommodate a dual lumen balloon. Derivo 2 is a 48-nitinol composite wire construct with a high platinum core stent. Its construction is designed to make the stent open smoothly and adapt to the vessel walls. In both of these cases, we encountered significant friction of the stent inside the microcatheter due to poor trackability. As a result, not only did the intermediate catheter need to be parked quite distally (i.e., at the origin of A1), but in the second case, the balloon had also to be navigated into the A4 segment, to allow the stent to take the A1–A2 curve despite otherwise straightforward anatomy with little tortuosity.

We acknowledge that simple coiling could have been considered for the two cases, nevertheless, the use of remodeling technique in experienced centers has proven not to increase procedural risks and we prefer using the balloon also because it can be inflated if an aneurysmal rupture occurs (4).

The technique we illustrate has only been published for the treatment of proximal aneurysms with 21 inch compatible FDs and it merges the advantages of balloon assisted coiling and flow-diversion (5). Low-profile FDs might therefore replace braided stents in the so-called “balloon-then-stent” technique, especially in cases of bifurcation aneurysms (6).

Stent deployment following balloon-assisted coiling is a well-described technique, but there are no reports on this technique using low-profile flow-diverters to our knowledge (7).

## Learning points/take home messages

- Flow diverters deployment following balloon-remodeling is a promising technique for challenging bifurcation aneurysms.
- Large diameter flow diverter stents still do not fit 0.017 microcatheters.
- The Derivo 2 can be deployed through a Scepter C balloon, but trackability is poor and tortuous anatomy may present a major obstacle.

## Data availability statement

The original contributions presented in the study are included in the article/supplementary material, further inquiries can be directed to the corresponding author.

## Ethics statement

Written informed consent was obtained from the individuals for the publication of any potentially identifiable images or data included in this article.

## Author contributions

LR and FC performed the cases and wrote the draft. SN, AL, and CC reviewed the literature and reviewed the manuscript. NL wrote the draft and reviewed the manuscript.
